# Supplementary material for: Light from van der Waals quantum tunneling devices
Source: Nat Commun. 2019 Jan 17;10:292. doi: 10.1038/s41467-018-08266-8 (PMC6336876; doi:10.1038/s41467-018-08266-8)
Supplement: Supplementary file 1 — Supplementary Information [file 41467_2018_8266_MOESM1_ESM.pdf]

— **Supplementary Information** —

Light from van der Waals quantum tunneling devices

Parzefall *et al.*

Supplementary Note 1: Tunneling Characteristics

A vertical cross-section of a vdWQT device is shown in Supplementary Figure 1a. Applying a voltage V_b between gold and graphene generates a tunnel current through the insulating h-BN. The measured voltage-dependent current density for a six-layer h-BN device is shown in blue in Supplementary Figure 1c. As expected, the tunnel current varies non-linearly with applied bias. Furthermore, in the voltage range $|V_\mathrm{b}| < 0.5\,\mathrm{V}$ some irregularities are visible. These minute features are pronounced more strongly when analyzing the first derivative of the tunneling current with respect to the applied bias ($\mathrm{d}I/\mathrm{d}V$), cf. red curve in Supplementary Figure 1c. Three distinct minima are visible.

The central $\mathrm{d}I/\mathrm{d}V$ minimum is indicative of the presence of a phonon-induced tunneling channel as originally observed in scanning tunneling microscopy experiments [1, 2]. Graphene's electronic states associated with the K and K' points of reciprocal space are characterized by a large in-plane momentum. As a consequence, direct tunneling into and from these states is weak because of the momentum mismatch with states in the gold electrode, as well as a faster decay of the local density of electronic states in transport direction [3], which is evident from the low conductivity around zero bias. At applied voltages $V_\mathrm{b} \geq \hbar\Omega/e$, where $\hbar\Omega = 63\,\mathrm{meV}$ [2] is the phonon energy ($\hbar$ is the reduced Planck constant, Ω is the phonon frequency and e is the elementary charge), an additional tunneling channel opens up that couples electronic states at the K/K' points with states at the Γ point. This onset of phonon-assisted tunneling causes a sudden increase in conductivity. Consequently, the $\mathrm{d}I/\mathrm{d}V$ exhibits a minimum at zero bias whose width is given by twice the phonon energy $\hbar\Omega$.

Both $\mathrm{d}I/\mathrm{d}V$ minima at negative and positive voltages are caused by the minimum of graphene's electronic density of states [4]

$$\rho_\mathrm{Gr}\left(E\right) = \frac{2\left|E - E_\mathrm{D}\right|}{\pi\hbar^2 v_\mathrm{F}^2}, \tag{1}$$

at the Dirac point, where v_F is the Fermi velocity of graphene. The location of the minima is determined by the electrostatics of the heterostructure. As described in the Methods section of the main text, in addition to serving as a tunnel contact, the gold electrode acts as an electrostatic gate, changing the charge carrier density n_Gr and consequently the Fermi level position E_F of the graphene sheet with respect to the Dirac point energy E_D as $\Delta E_\mathrm{F} = E_\mathrm{F} - E_\mathrm{D}$. This dual functionality is illustrated in the band alignment diagrams shown in Supplementary Figure 1b. Differences in the band alignment and dipole layers at the gold / h-BN and graphene / h-BN

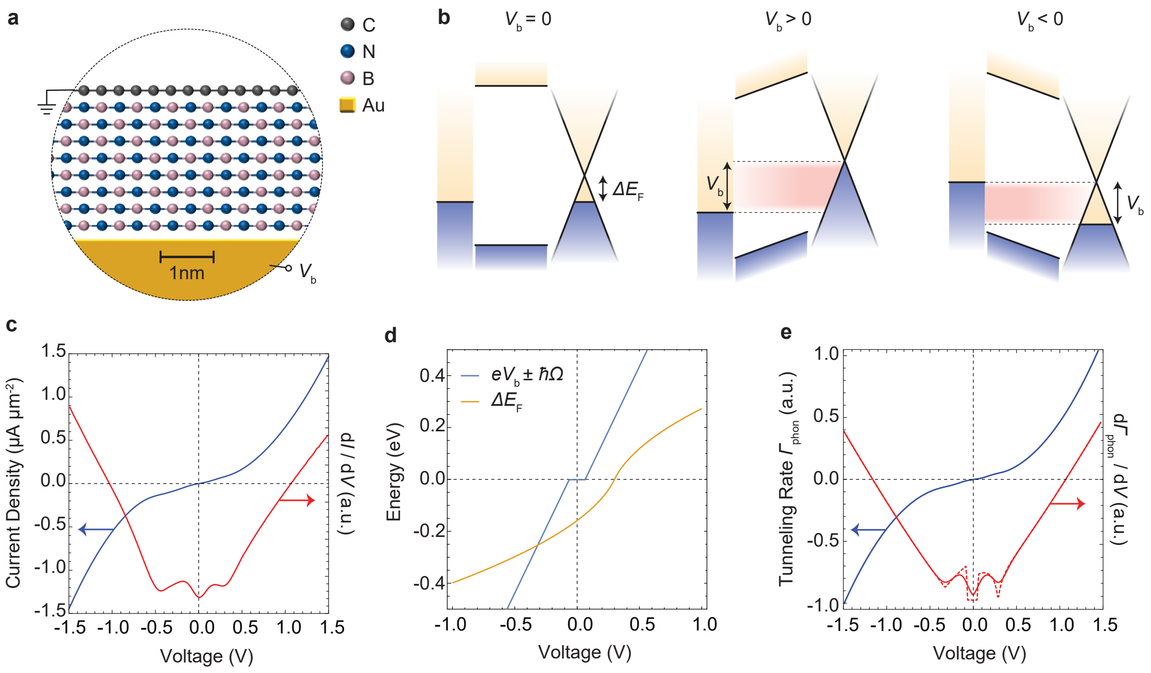

Supplementary Figure 1. Electronic properties of vdWQT devices. **a** Schematic of the vertical configuration of the device, i.e. gold–few-layer h-BN–graphene. A voltage V_{b} is applied between the gold and graphene electrodes. **b** Band alignment diagrams at zero bias (left), positive bias (center) and negative bias (right). ΔE_{F} marks the shift of the graphene Fermi level with respect to the Dirac point. Areas shaded in blue, yellow and red mark energetic regions of occupied states, unoccupied states and tunneling through h-BN, respectively. **c** Measured current-voltage characteristics and its first derivative of a gold–6L h-BN–graphene device. The derivative displays three distinct minima. **d** Effective applied voltage, i.e. voltage reduced by the phonon energy $\hbar\Omega$, and the calculated, electrostatically induced shift in the graphene Fermi level ΔE_{F} as a function of applied voltage for $V_0 = 0.3\,\mathrm{eV}$. **e** Calculated voltage-dependence of the phononic tunneling rate Γ_{phon} and its first derivative of a gold–6L h-BN–graphene device at 0 K (dashed lines) and 300 K (solid lines).

interfaces cause initial charge equilibration, which leads to a finite charge carrier density n_0 at $V_{\mathrm{b}} = 0$ and a corresponding shift ΔE_{F} of the Fermi level away from the charge neutrality point. For our devices we find that $\Delta E_{\mathrm{F}}(V_{\mathrm{b}} = 0) < 0$, i.e. the graphene sheet is hole-doped at zero bias (cf. left diagram of Supplementary Figure 1b). The calculated dependence of ΔE_{F} on applied voltage is shown in Supplementary Figure 1d for an offset voltage of $V_0 = 0.3\,\mathrm{eV}$ (cf. Methods). For positive voltages, the initial doping is reduced with increasing voltage until charge neutrality is reached at $V_{\mathrm{b}} = V_0$ such that $\Delta E_{\mathrm{F}}(V_{\mathrm{b}} = V_0) = 0$ [5], as shown in the center of Supplementary Figure 1b. The value of V_0 determines the location of the first $\mathrm{d}I/\mathrm{d}V$ minimum (here at positive voltages).

The second minimum is reached at negative voltages when the applied voltage (reduced by the phonon energy $\hbar\Omega$) equals the Fermi level offset ΔE_{F}, as illustrated on the right of Supplementary Figure 1b.

We model the different tunneling channels present in vdWQT devices within the framework of the transfer Hamiltonian formalism [6, 7]. Here, the rate of elastic tunneling[1] is given by[2]

$$\Gamma_{\mathrm{el}} = \frac{2\pi}{\hbar} \int_0^{eV_{\mathrm{b}}} |\mathcal{T}(E)|^2 \rho_{\mathrm{Au}}(E)\rho_{\mathrm{Gr}}(E)\,\mathrm{d}E, \tag{2}$$

where $\mathcal{T}(E)$ is the transfer Hamiltonian matrix element (cf. Supplementary Note 2), and ρ_{Au} is the electronic density of states of gold. As discussed previously, tunneling in metal–insulator–graphene devices is dominated by phonon-enabled inelastic tunneling. We describe the corresponding 'phononic' tunneling rate Γ_{phon} in analogy to elastic tunneling as

$$\Gamma_{\mathrm{phon}} = \xi \frac{2\pi}{\hbar} \int_{\hbar\Omega}^{eV_{\mathrm{b}}} |\mathcal{T}(E)|^2 \rho_{\mathrm{Au}}(E-\hbar\Omega)\rho_{\mathrm{Gr}}(E)\,\mathrm{d}E, \tag{3}$$

where ξ denotes the probability ratio between elastic tunneling and phononic inelastic tunneling. The equation as stated applies to the positive voltage range. For negative voltages, the roles of the two electronic state densities are interchanged. Here, in contrast to equation (2), the 'activation energy' required for phonon-induced tunneling is accounted for by restricting the integration interval to $|eV_{\mathrm{b}}| - \hbar\Omega$. Furthermore, since a tunneling event is accompanied by the creation of a phonon of energy $\hbar\Omega$, equation (3) connects electronic states of different energies. While ρ_{Gr} is given by equation (1), ab initio device simulations based on density functional theory (DFT) suggest that both $\mathcal{T}$ and ρ_{Au} depend only weakly on energy and are hence taken to be constant, cf. Supplementary Note 3. The corresponding results of equation (3) are shown in Supplementary Figure 1e. We find good agreement between theory and experiment. The minima of the $\mathrm{d}I/\mathrm{d}V$ are reproduced by our model,[3] their location corresponds to the points where $\Delta E_{\mathrm{F}} = 0$ as well as where $|eV_{\mathrm{b}}| - \hbar\Omega = |\Delta E_{\mathrm{F}}|$, cf. Supplementary Figure 1d.

We now turn to the relative contribution of the different tunneling channels to the overall

---

[1] By elastic tunneling we refer to tunneling processes in which the energy of the electron remains unchanged.

[2] For brevity we state equations in the limit $k_{\mathrm{B}}T \to 0$ and omit Fermi-Dirac distribution functions.

[3] Thermal broadening cannot fully account for the measured width of the $\mathrm{d}I/\mathrm{d}V$ minima compared to model calculations for $0\,\mathrm{K}$ (dashed line in Supplementary Figure 1e). The additional broadening varies from device to device as well as with device size, and is caused by fluctuations in the charge density of graphene due to residual contaminants [8, 9]. We account for this additional broadening by assuming a normal distribution of V_0 with a width of $\sim 50\,\mathrm{meV}$.

tunneling rate Γ. It can be expressed as a sum of three tunneling rates as

$$\Gamma = \Gamma_{\mathrm{el}} + \Gamma_{\mathrm{phon}} + \hbar \int_0^\infty \gamma_{\mathrm{phot}}(\hbar\omega)\mathrm{d}\omega. \tag{4}$$

Our theoretical analysis of the tunneling characteristics suggests that the tunneling current is dominated by the phonon-enhanced tunneling channel Γ_{phon} as it efficiently bridges the momentum mismatch between the two electrodes. This finding stands in agreement with previous experimental and theoretical works [1–3, 9]. The spectral rate $\gamma_{\mathrm{phot}}(\hbar\omega)$ shown in Figure 4c/g is normalized to the elastic tunneling rate Γ_{el}. This normalization reveals that the quantity $\gamma_{\mathrm{phot}}(\hbar\omega)/\Gamma_{\mathrm{el}}$ is of the order of $2 \times 10^{-7}\,\mathrm{eV}^{-1}$ in the uncoupled vdWQT. The experimentally measured value for the spectral efficiency (Figure 4a/e), which is of the order of $5 \times 10^{-9}\,\mathrm{eV}^{-1}$, is a measure for $\gamma_{\mathrm{phot}}(\hbar\omega)/\Gamma \approx \gamma_{\mathrm{phot}}(\hbar\omega)/\Gamma_{\mathrm{phon}}$. A comparison of these values allows us to estimate that $\Gamma_{\mathrm{phon}} \sim 40 \times \Gamma_{\mathrm{el}}$, which is of the same order of magnitude as the value reported by Zhang et al. [2].

Supplementary Note 2: Transfer matrix elements

The transfer matrix element $\mathcal{T}(E)$ is given by [7]

$$\mathcal{T}(E) = \frac{\hbar^2}{2m}\left[\psi_{\mathrm{Au}}\frac{\mathrm{d}\psi^*_{\mathrm{Gr}}}{\mathrm{d}z} - \psi^*_{\mathrm{Gr}}\frac{\mathrm{d}\psi_{\mathrm{Au}}}{\mathrm{d}z}\right]_{z=z_0}. \tag{5}$$

The momentum matrix element $\mathcal{P}(E)$ on the other hand is given by the expectation value of the momentum operator $\hat{p}_z = -\mathrm{i}\hbar\,\mathrm{d}/\mathrm{d}z$ and reads as

$$\mathcal{P}(E, \hbar\omega) = \langle\psi_{\mathrm{Au}}\left|\hat{p}_z\right|\psi_{\mathrm{Gr}}\rangle = -\mathrm{i}\hbar\int_0^{d_{\mathrm{hBN}}}\psi^*_{\mathrm{Au}}(E-\hbar\omega)\frac{\mathrm{d}}{\mathrm{d}z}\psi_{\mathrm{Gr}}(E)\,\mathrm{d}z. \tag{6}$$

The equation as stated applies to the positive voltage range as it connects initial states in the graphene electrode to final states in the gold electrode at an energy difference given by $\hbar\omega$, cf. Figure 4b. For negative voltages, the roles of the two electronic wave functions are interchanged.

To calculate the matrix elements we need to mathematically describe the electronic wavefunction inside the h-BN band gap. Within the transfer Hamiltonian framework, these wavefunctions are approximately given by the wavefunction of the individual electrodes in the absence of the other electrode, respectively. Inside the band gap of h-BN, gold and graphene wave functions $\psi_{\mathrm{Au}}(z)$ and $\psi_{\mathrm{Gr}}(z)$, respectively, decay exponentially with distance from the interface as:

$$\begin{aligned}\psi_{\mathrm{Au}}(z) &= \psi_{\mathrm{Au},0}\,\mathrm{e}^{-\kappa z}, \quad z \geq 0\\ \psi_{\mathrm{Gr}}(z) &= \psi_{\mathrm{Gr},0}\,\mathrm{e}^{-\kappa(d_{\mathrm{hBN}}-z)}, \quad z \leq d_{\mathrm{hBN}}\end{aligned} \tag{7}$$

For simplicity we assume the decay constant κ to be independent of z and energy E (cf. Supplementary Note 3) which results in the following expressions for the matrix elements:

$$\begin{aligned}\mathcal{T} &= \frac{\hbar^2\kappa}{m}\psi_{\mathrm{Au},0}\,\psi_{\mathrm{Gr},0}\,\mathrm{e}^{-\kappa d_{\mathrm{hBN}}}\\ \mathcal{P} &= -\mathrm{i}\hbar\kappa d_{\mathrm{hBN}}\,\psi_{\mathrm{Au},0}\,\psi_{\mathrm{Gr},0}\,\mathrm{e}^{-\kappa d_{\mathrm{hBN}}}\end{aligned} \tag{8}$$

Supplementary Note 3: Ab initio device simulation

To examine the energy-dependence of the decay constant κ we performed atomistic transport simulations of the vdWQT device structure based on density-functional theory (DFT). Supplementary Figure 2a shows an atomistic representation of the heterostructure. The unit cell of the heterostructure with all three materials present, that is, the middle part of Supplementary Figure 2a, is simulated by the ab initio DFT tool VASP [10] within the generalized gradient approximation of Perdew, Burke, and Ernzerhof [11]. Van der Waals interactions are included through the DFT-D2 method of Grimme [12]. After the geometric optimization of the ions the single-particle electron states are

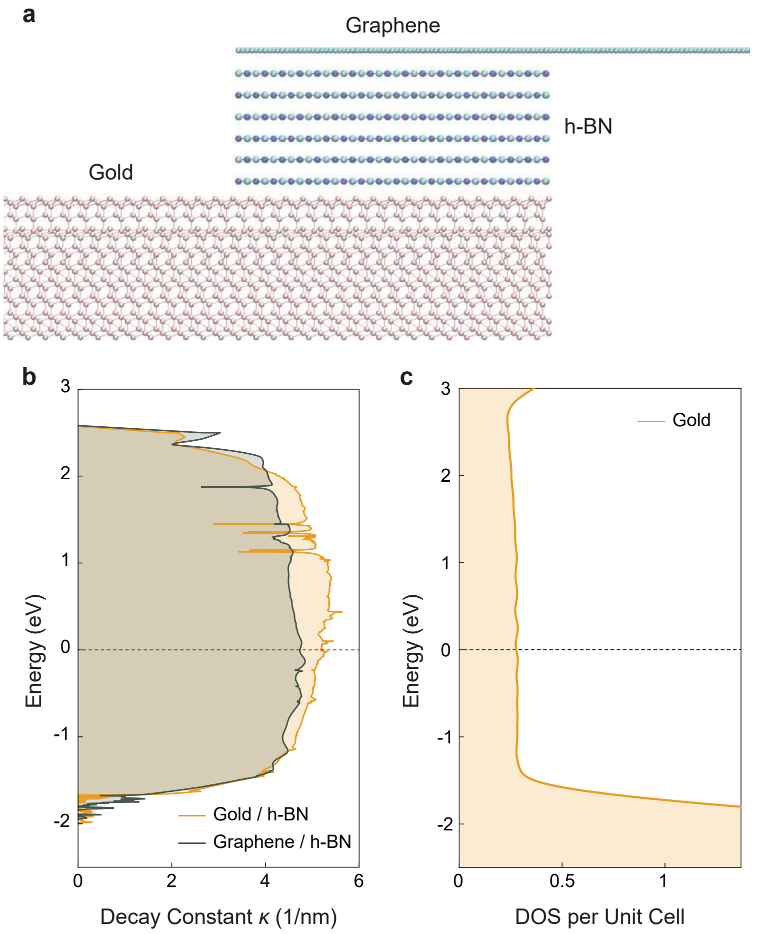

Supplementary Figure 2. DFT of vdWQT devices. **a** Atomistic representation of the simulated heterostructure consisting of gold, h-BN and graphene. **b** Calculated values for the decay constant κ, extracted from the exponential decay of the DOS within h-BN as a function of distance from graphene/gold, plotted as a function of electron energy. The zero of the vertical energy axis is aligned with the Fermi level in gold . **c** Calculated density of states per unit cell inside the gold electrode.

determined and transformed into a set of maximally localized Wannier functions (MLWFs) with the use of the Wannier90 tool [13]. The Hamiltonian of the device depicted in Supplementary Figure 2a is built up from the MLWF matrix elements following the procedure described in Appendix B of ref [14]. The resulting Hamiltonian is loaded into a quantum transport simulator based on the non-equilibrium Green's function (NEGF) formalism [15]. The energy-resolved local density of electronic states (DOS) is determined at a constant zero external potential without considering self-consistency. In the middle, insulating part of the device, an exponential curve is fitted to the position-dependent DOS at every energy point, separately for the left- and right-injected states, corresponding to the gold and the graphene states, respectively. Since the DOS is proportional to the square of the wavefunction, the κ decay rate is calculated as one half of the characteristic length of the fitted exponential curves. The resulting values for κ are shown in Supplementary Figure 2b. We find that κ is similar for both interfaces and only weakly depends on energy across the band gap except close to the h-BN conduction and valence band edges at approximately $-1.7\,\mathrm{eV}$ and $+2.1\,\mathrm{eV}$, respectively. As expected, the simulation underestimates the band gap of h-BN. These results suggest that for our devices $\kappa d_{\mathrm{hBN}} \gg 1$, hence we conclude that we operate well within the validity limits of the transfer-Hamiltonian formalism [7].

We further calculate the density of electronic states $\rho_{\mathrm{Au}}(E)$ within the unit cell of bulk gold by VASP, shown in Supplementary Figure 2c. The electronic DOS of gold is found to be approximately constant over the relevant energy range.

Supplementary Note 4: Local Density of Optical States

Uncoupled vdWQT devices. We are interested in the partial LDOS along the direction of electron flow and hence consider the dipole orientation $\mathbf{p} = p_z\mathbf{z}$, where $\mathbf{z}$ is the unit vector perpendicular to the heterostructure. The power P dissipated by a dipole placed at $\mathbf{r}_0$ and oscillating at frequency ω is given by [16]

$$P = \frac{\omega}{2}\mathrm{Im}\left\{\mathbf{p}^* \cdot \mathbf{E}(\mathbf{r}_0)\right\} = \frac{\omega}{2}p_z\mathrm{Im}\left\{E_z(\mathbf{r}_0)\right\}, \tag{9}$$

where $\mathbf{E}(\mathbf{r}_0)$ is the electric field at the position of the dipole. This field is a superposition of the primary dipole field $\mathbf{E}_0(\mathbf{r}_0)$ and the secondary field $\mathbf{E}_\mathrm{s}(\mathbf{r}_0)$ that is a result of the interaction with the environment. The total field reads as

$$\mathbf{E}(\mathbf{r}_0) = \mathbf{E}_0(\mathbf{r}_0) + \mathbf{E}_\mathrm{s}(\mathbf{r}_0). \tag{10}$$

The different modes of a layered system are distinguishable in terms of their in-plane momentum $k_{||}$. For photons, $k_{||}$ is given by the projection of the wave vector k onto the heterostructure plane. In the case of guided modes, it corresponds to the complex propagation constant of the mode. Hence it is useful to express the electric field in terms of a superposition of all possible $k_{||}$. This 'angular spectrum representation' of the z-component of the primary dipole field, in cylindrical coordinates and at $r = r_0$, can be derived as [16]

$$E_z(z, z_0) = \frac{\mathrm{i}\omega^2 p_z}{4\pi c^2 \varepsilon_0 \varepsilon_i}\int_0^\infty \frac{k_{||}^3}{k_i^2 k_{zi}}\mathrm{e}^{\mathrm{i}k_{zi}|z-z_0|}\mathrm{d}k_{||}, \tag{11}$$

where ε_0 is the vacuum permittivity and ε_i is the dielectric permittivity of the medium surrounding the dipole. We additionally introduce the normalized variables $s = k_{||}/k_0$, where k_0 is the wave vector in vacuum, and $k_{zi} = k_0\sqrt{n_i^2 - s^2} = k_0 s_{zi}$, where n_i is the refractive index of the medium surrounding the dipole, such that equation (11) becomes

$$E_z(z, z_0) = \frac{\mathrm{i}\omega^3 p_z}{4\pi\varepsilon_0\varepsilon_i c^3}\int_0^\infty \frac{s^3}{s_{zi}}\mathrm{e}^{\mathrm{i}k_0 s_{zi}|z-z_0|}\mathrm{d}s. \tag{12}$$

For the simplest case of a dipole radiating in vacuum, the evaluation of equation (9) using equation (12) yields the familiar result

$$P_0 = \frac{\omega^4 p_z^2}{12\pi\varepsilon_0 c^3}. \tag{13}$$

In vdWQT devices, the 'dipole' is embedded in a layered medium with interfaces above and below that give rise to a secondary field $\mathbf{E}_\mathrm{s}(\mathbf{r}_0)$. Equation (12) allows us to decompose the field

generated by the dipole into a superposition of plane and evanescent waves. For a source embedded in a layered medium consisting of N layers, situated in layer i at the position z_0, the z-variation of the electric field in layer j can be expressed as [17]

$$\mathcal{F}_j(z, z_0) = \delta_{ij} \mathrm{e}^{\mathrm{i}k_{zi}|z-z_0|} + c_j^{\uparrow} \mathrm{e}^{\mathrm{i}k_{zj}z} + c_j^{\downarrow} \mathrm{e}^{-\mathrm{i}k_{zj}z}, \tag{14}$$

where $c_j^{\uparrow}$ and $c_j^{\downarrow}$ are complex field amplitudes of upward and downward propagating waves, respectively. Hence, the z-component of the electric field of a dipole embedded in a layered medium, at the position of the dipole $z = z_0 = 0$,[4] follows from equations (12) and (14) as

$$E_z = \frac{\mathrm{i}\omega^3 p_z}{4\pi\varepsilon_0\varepsilon c^3} \int_0^{\infty} \frac{s^3}{s_{zi}} \left(1 + c_i^{\uparrow} + c_i^{\downarrow}\right) \mathrm{d}s, \tag{15}$$

which in combination with equations (9) and (13), yields the following expression for the normalized dissipated power or, equivalently, the normalized LDOS:

$$\frac{\rho_{\mathrm{opt}}}{\rho_0} = \frac{P}{P_0} = \frac{3}{2} \int_0^{\infty} \mathrm{Re}\left\{ \frac{s^3}{s_{zi}\varepsilon_i} \left(1 + c_i^{\uparrow} + c_i^{\downarrow}\right) \right\} \mathrm{d}s \tag{16}$$

The complex field amplitudes $c_j^{\uparrow}$ and $c_j^{\downarrow}$ are calculated by applying the boundary conditions for p-polarized fields to equation (14) [16]. The presence of graphene is included by a surface charge density at the respective interface through the boundary conditions [18]. Graphene's in-plane conductivity is calculated within the local random-phase approximation (RPA) at $T = 300\,\mathrm{K}$ as described in ref [19].

Equation (16) allows for the calculation of the angular spectrum of the normalized LDOS of an arbitrary, layered geometry. The resulting spectrum for a Air–Graphene–h-BN–Gold–Glass[5] stack, where the dipole is located in the center of the h-BN domain, is shown in Supplementary Figure 3a. Two peaks are visible at $s \sim 1.02$ and $s \sim 1.6$, corresponding to the two SPP modes at the top and bottom surface of the gold electrode, respectively. Supplementary Figure 3b-d shows detailed excerpts of the angular spectrum corresponding to direct photon emission into angles that are detectable through a NA = 0.9 objective (b) and the two SPP modes (c,d). The former is associated with the (detectable) radiative LDOS ρ_{rad} of the uncoupled, planar heterostructure, responsible for the experimentally observed direct photon emission. For comparison we carried out calculations with and without graphene (straight and dashed lines, respectively). As discussed

[4] Without loss of generality we set $z_0 = 0$. The case of $z_0 \neq 0$ introduces a global phase that does not influence the results of the calculation.

[5] The values of the individual refractive indices and dielectric functions are taken from literature as discussed in Supplementary Note 4.

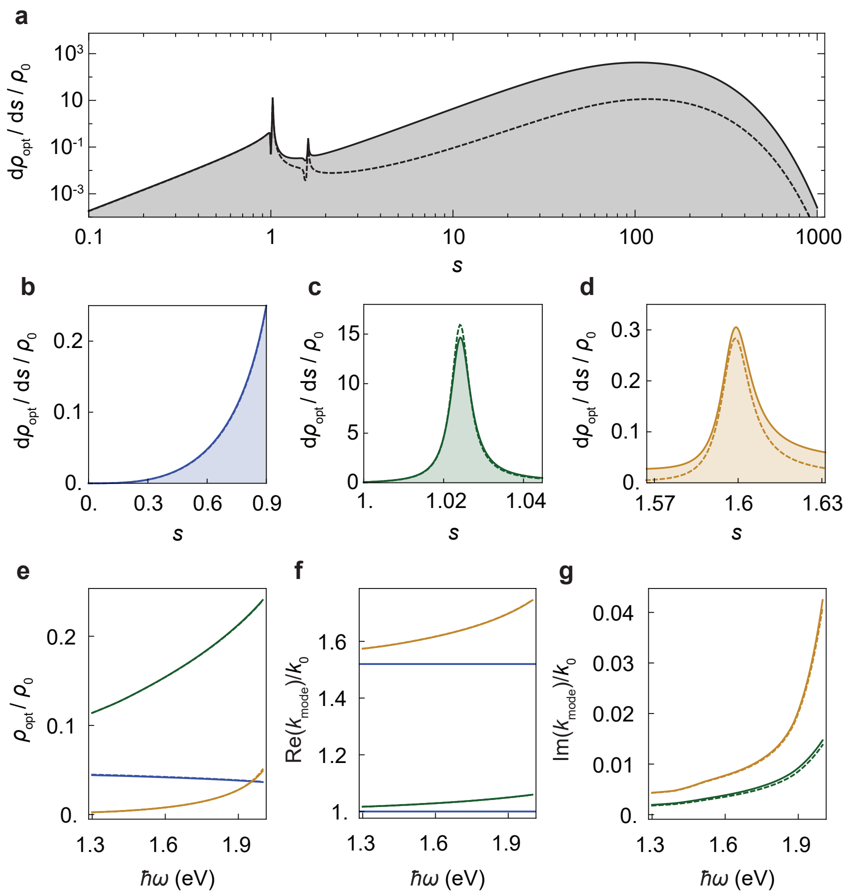

Supplementary Figure 3. Optical properties of a Air–Graphene–h-BN ($7 \times 0.33\,\mathrm{nm}$)–Gold ($50\,\mathrm{nm}$)–Glass stack. Straight and dashed lines refer to calculations with and without graphene, respectively. **a** Angular spectrum of the normalized LDOS $\rho_{\mathrm{opt}}/\rho_0$, calculated at an energy of $1.5\,\mathrm{eV}$, for a dipole oriented perpendicular to the plane and placed in the center of the h-BN. **b-d** Excerpts of the LDOS spectrum showing the regions related to (collectible) photons (**b**) and the two SPP modes associated with the top (**c**) and bottom (**d**) surface of the gold film. **e** LDOS for photons (blue), top SPPs (green) and bottom SPPs (orange) as a function of mode energy. The LDOS of the SPP modes is extracted by fitting a Lorentzian on top of a linear background. The area of the curve corresponds to the LDOS of the mode. **f,g** The center and FWHM of the Lorentzian correspond to the real (**f**) and imaginary (**g**) part of the propagation constant k_{mode}, respectively, shown for the top (green) and bottom SPPs (orange). The blue lines in **f** correspond to the refractive indices of air ($n = 1$) and glass ($n = 1.52$).

in the main text, since the primary field components of the SPP modes are perpendicular to the heterostucture plane, the modes only weakly interact with the graphene. Supplementary Figure 3a shows that the presence of graphene—in the energy-range of interest—primarily causes additional quenching, evidenced by the increase of the LDOS for large values of s.

To analyze the dependence on mode energy we fit Lorentzian line-shapes (plus a linear

background) to the calculated angular spectra at each energy and extract the LDOS of the mode by calculating the area of the Lorentzian. The LDOS for photon emission is calculated by numerically integrating over the angular spectrum shown in Supplementary Figure 3b. The resulting energy-dependence is shown in Supplementary Figure 3e. The LDOS of the two SPP modes increases with energy due to the increase in mode confinement and propagation constant. On the other hand, the photon LDOS remains approximately constant at $\sim 0.04 \times \rho_0$.[6] Furthermore, the LDOS of all modes is of the same order of magnitude. Additionally we extract the real and imaginary parts of the propagation constant of the two SPP modes as the center and FWHM of the Lorentzian fits, respectively. The resulting energy-dependent values are shown in Supplementary Figure 3f/g, where the real part renders the dispersion relation of the two modes and the imaginary part determines its field propagation length as $1/\mathrm{Im}(k_{\mathrm{mode}})$. Again we find that the graphene sheet has a negligible effect on the propagation constant of these modes.

We note that our geometry supports graphene plasmons in the infrared spectral range that are not discussed here but that can be excited by electron tunneling. These plasmons do not affect our results as they are strongly damped for energies $\hbar\omega > 2\Delta E_{\mathrm{F}}$ [18]. As the maximum ΔE_{F} reached in our experiments is $\sim 0.45\,\mathrm{eV}$ (cf. Figure 4d/h), graphene plasmons do not contribute to the signal in our detection window. The exploration of the excitation of these plasmons by inelastic electron tunneling is particularly interesting in graphene–insulator–graphene tunneling devices [20–22].

---

[6] The apparent suppression of the radiative LDOS is the result of the high-index medium (h-BN) at the emitter position, which leads to a redistribution of the LDOS towards higher values of s, as the plane wave region in this case extends up until $s = n_{\mathrm{hBN}}$, where n_{hBN} is the refractive index of h-BN. While (in a homogeneous medium of refractive index n) the overall plane wave LDOS increases linearly with n, the fraction that is emitted into the angular range detectable in our experiment, given by $0 \leq s \leq \mathrm{NA}$, decreases, cf. equation (16). This effect is responsible for the low value of ρ_{rad}.

Antenna-coupled vdWQT devices. To determine the LDOS inside vdWQT device-coupled nanocube antennas we perform numerical finite element simulations using the wave optics module of the commercially available software package COMSOL 5.3. To acquire the simulation results shown in Figure 5 we place an out-of-plane (in the direction of electron tunneling) oriented electric point dipole into the center of a 7×0.33 nm thick h-BN domain which is embedded between 50 nm of gold below and graphene on top. Graphene is modeled as a surface current density [23], calculated within the local random phase approximation (RPA) [19]. A 75 nm silver cube with an edge radius of 7.5 nm that is surrounded by a 3 nm thick PVP film is placed on top. The upper and lower half-spaces are air and glass, respectively, and perfectly matched layers are used at the boundaries of the simulation domain. The refractive indices n of the dielectric materials are $n_{\mathrm{glass}} = 1.52$, $n_{\mathrm{hBN}} = 2.2/2.0$ (parallel / perpendicular to the plane) [24] and $n_{\mathrm{PVP}} = 1.52$ [25]. Interpolated, empirical values are used for the dielectric functions of gold [26] and silver [27]. The radiative LDOS in units of ρ_0 is extracted as the power radiated into the air half-space above the antenna geometry by integrating the time-averaged Poynting vector over the simulation boundaries, normalized by the power radiated by the same dipole in vacuum as given by equation (13).

Supplementary Note 5: Nanocube Antennas

Cube-to-cube variations. The spatial intensity distribution of light emission shown in Figure 5d is characterized by four dominant emission spots corresponding to four individual nanocube antennas. Supplementary Figure 4a shows the emission spectra of these four cubes.[7] We find that the antennas resonantly enhance photon emission at slightly different energies. Supplementary Figure 4b shows scanning electron microscope (SEM) images of the vdWQT devices coupled to nanocube antennas. The overview image on the left shows the locations of the individual cubes. For the chosen SEM parameters the stack of two-dimensional materials does not generate a significant contrast and hence we only see the gold electrodes, the glass substrate and the silver nanocubes. The image suggests that a total of seven cubes is placed on top of the vdWQT devices. On the right we see magnified images of the individual cubes. The colored frames around the SEM images assign the cubes to their respective spectra in Supplementary Figure 4a.

The size of the nanocubes **4**, **6** and **5**, as extracted from SEM images, are 75 nm, 72 nm and 68 nm, respectively. With decreasing size, at constant gold-silver distance, the MIM resonator length decreases with decreasing cube size. This is expected to be accompanied by a blue-shift, i.e. a shift towards higher energies, of the lowest order resonator mode. This expectation is in agreement with our experimental observation of the different resonance energies shown in Supplementary Figure 4a. Antenna **3** on the other hand does not follow this trend with a size of 77 nm. Taking a closer look at the location of the cube we find that only part of the nanocube overlaps with the gold electrode, leading to an artificially shorter MIM resonator length and hence an additional shift towards higher energies. Due to its size of 57 nm we do not excite the fundamental resonance of cube **1** at the applied voltage of 2 V and hence observe no emission. We do observe weak emission from the remaining cubes **2** and **7** as seen in 5d, however we did not characterize their emission spectra. Some variation in intensity amongst antennas is to be expected and most likely caused by an imperfect attachment of the cube to the surface or residues from device fabrication that may either locally suppress electron tunneling if they are localized between the layers constituting the vdWQT device or shift the resonance energy out of our detection window, which is restricted to approximately $1.1\,\mathrm{eV} \leq \hbar\omega \leq |eV_\mathrm{b}|$, determined by the silicon band gap on the low end and the quantum cutoff condition on the high end.

---

[7] Please not that the EMCCD image shown in 5d is not corrected for spectral variations in detection efficiency and chromatic aberrations, leading to an apparent discrepancy in brightness compared to the fully corrected spectra shown in Supplementary Figure 4a.

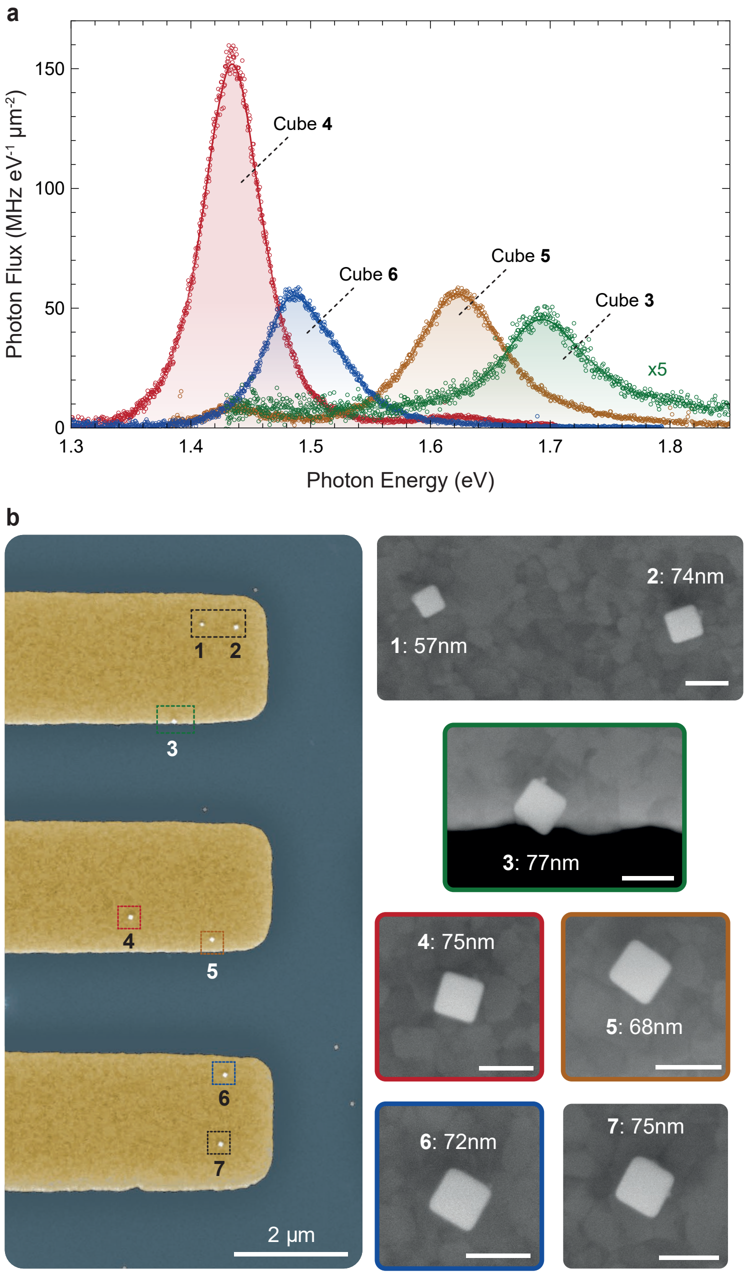

Supplementary Figure 4. Nanocube antennas driven by a vdWQT device. **a** Emission spectra of the four nanocube antennas that are clearly visible in Figure 5d at an applied voltage $V_b = 2.0\,\mathrm{V}$. **b** Scanning electron microscopy images of the three electrodes. The left side shows a false-color image of all three electrodes, showing the location of the different nanocubes. All nanocubes that are located on top of the vdWQT devices are numerated. The right side shows magnified images of the individual cubes, all scale bars 100 nm. The colored frames assign the cubes to their respective emission spectrum.

Voltage-dependence of antenna-coupled emission. We further analyze the voltage-dependence of antenna-coupled emission spectra, shown in Supplementary Figure 5. Panel a exemplarily shows emission spectra of Cube **4** (cf. Supplementary Figure 4) in the relevant positive (right) and negative (left) voltage ranges in steps of 0.2 eV. As only modes of energy $\hbar\omega < |eV_\mathrm{b}|$ can be excited, the resonance peak of Cube **4** at 1.43 eV is not visible at ± 1.3 V and emerges at higher voltages. For comparison we show the emission spectrum of the uncoupled heterostructure in Supplementary Figure 5a with a relative scaling factor of 2.8×10^4. In the main text we found that the local enhancement of the spectral photon emission rate closely corresponds to the calculated enhancement of the radiative LDOS. This implies that the enhancement should be independent of the applied voltage. To verify this we extract the peak spectral efficiencies of four cube antennas as a function of voltage as the amplitude of Lorentzian peak functions which we fit to the corresponding spectra. For comparison we extract the spectral efficiency of the unmodified heterostructure (cf. Figure 4a/e) at the energy value that corresponds to the resonance position of the cube antenna. The results are shown in Supplementary Figure 5b-e. Overall we find that indeed each cube can be characterized with a single peak enhancement factor that generates a direct, close to voltage-independent link to the emission efficiency of the planar heterostructure. Minor deviations are to be expected as the nanocube antenna probes an area that is three orders of magnitude smaller than the total area of the tunnel junction. Furthermore, the cubes may cause small changes to the local offset V_0 of the Fermi level position ΔE_F of the graphene sheet which is most likely responsible for the asymmetric deviation observed generally and particularly pronounced for cube **4**.

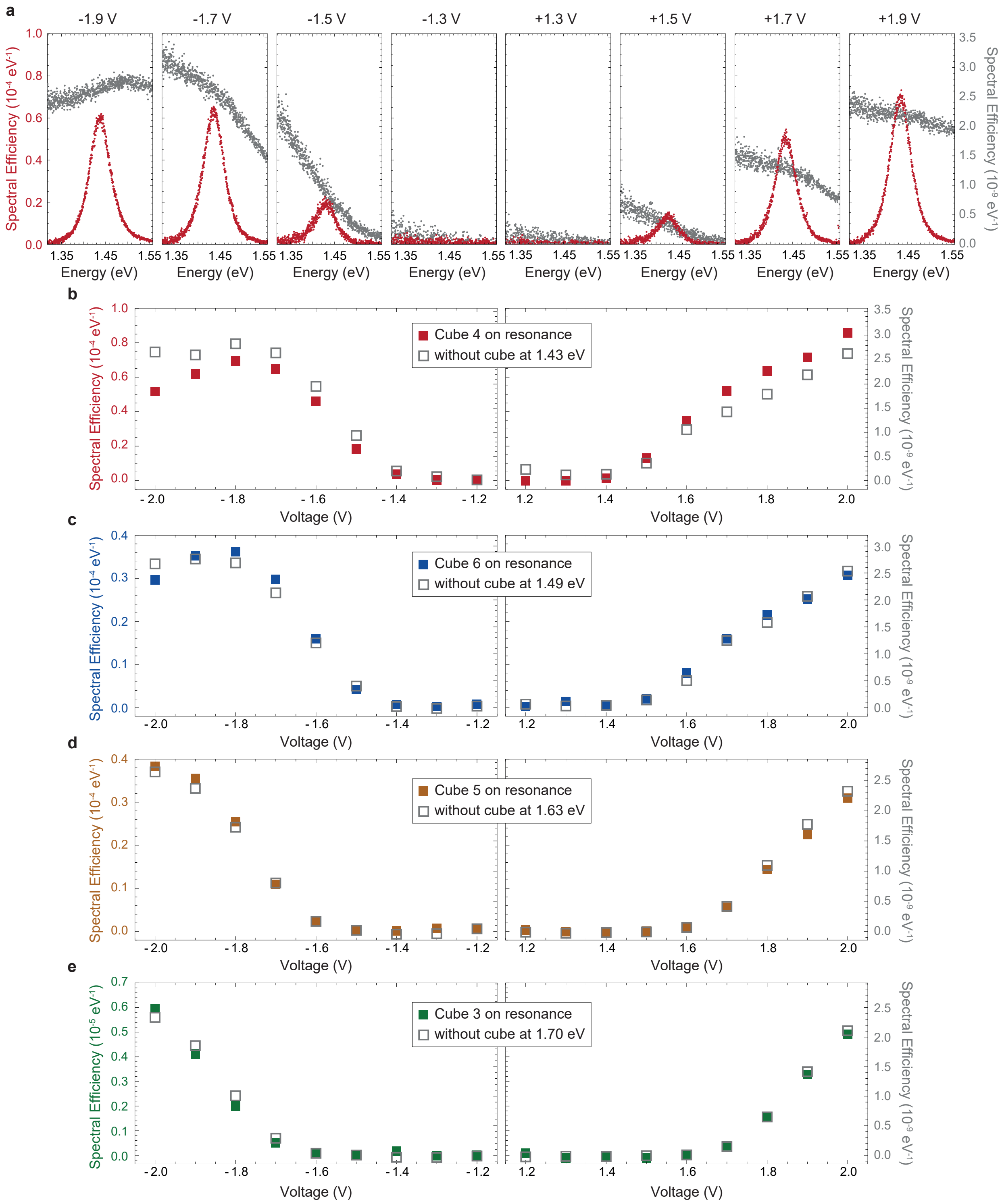

Supplementary Figure 5. Voltage-dependence of antenna-coupled emission. **a** Antenna emission spectra (Cube **4**) at several negative and positive voltage values. For comparison we show the emission spectrum of the planar heterostructure (cf. Figure 4a/e) within the emission window of the nanocube antenna. **b-e** Amplitude of the Lorentzian fit to the antenna-coupled emission spectra as a function of voltage in comparison to the spectral efficiency value of the planar heterostructure at the peak-value of the resonance for Cubes **4**, **6**, **5** and **3**, respectively.

SPP scattering by nanocube antennas. The scattering of SPPs that are excited by inelastic electron tunneling all across the tunnel junction area constitutes a secondary contribution to the observed, localized signal emerging from the cube antenna-coupled vdWQT devices. In the following we estimate the magnitude of this contribution.

The emission of cube **4** on resonance is enhanced by a factor of 3×10^4 (cf. Figure 5e) compared to the planar heterostructure, within the footprint of the antenna ($75 \times 75\,\mathrm{nm}^2$). On the other hand, the area of the center electrode is $\sim 4 \times 2\,\mu\mathrm{m}^2$ and hence $\sim 1.4 \times 10^3$ times larger than the active area of the nanocube antenna. Hence, the total spectral photon emission rate of the planar electrode is about a factor of 20 smaller than the spectral photon emission rate of the cube antenna on resonance. From this we may infer the ratio of photons emitted by the antenna to SPPs launched by the entire electrode by considering the respective LDOS contributions. As the LDOS of the SPP mode at the top interface is about a factor of three higher than the radiative LDOS (at 1.43 eV, cf. Supplementary Figure 3e) we conclude that the total SPP emission rate is about a factor of seven lower than the spectral photon emission rate of the antenna on resonance.

To estimate the fraction of launched SPPs that is scattered by a single nanocube antenna we carried out numerical finite element simulations (COMSOL 5.3) to determine the cube antennas SPP scattering cross section. As the SPP is confined along the out-of-plane direction we may define a scattering width σ_w to describe the efficacy of SPP scattering. This scattering width is determined by the power scattered by the nanocube antenna compared to the power per unit width injected into the system. Numerically (for the same geometry described in Supplementary Note 4) we find that the scattering width of the nanocube antenna on resonance is approximately half the cube edge length. Furthermore, the scattering efficiency η_sct of an SPP launched at any given position at a distance r from the nanocube is well approximated (if the junction dimensions are much larger than the cube size) by $\eta_\mathrm{sct} \sim \sigma_\mathrm{w}/(2\pi r)$. Assuming the nanocube to be located in the center of a $\sim 4 \times 2\,\mu\mathrm{m}^2$ (yielding the highest efficiency) large tunnel junction we find that the average scattering efficiency is less than 1 %. We can hence estimate that the emission rate due to SPP scattering is two to three orders of magnitude lower than the experimentally measured antenna-coupled photon emission rate and can thus be safely neglected.

Supplementary References

---

[1] Brar, V. W. *et al.* Scanning tunneling spectroscopy of inhomogeneous electronic structure in monolayer and bilayer graphene on sic. *Appl. Phys. Lett.* **91**, 122102 (2007).

[2] Zhang, Y. *et al.* Giant phonon-induced conductance and scanning tunneling spectroscopy of gate-tunable graphene. *Nature Phys.* **4**, 627–630 (2008).

[3] Wehling, T. O., Grigorenko, I., Lichtenstein, A. I. & Balatsky, A. V. Phonon-mediated tunneling into graphene. *Phys. Rev. Lett.* **101**, 216803 (2008).

[4] Castro Neto, A. H., Guinea, F., Peres, N. M. R., Novoselov, K. S. & Geim, A. K. The electronic properties of graphene. *Rev. of Mod. Phys.* **81**, 109–162 (2009).

[5] Bokdam, M., Khomyakov, P. A., Brocks, G., Zhong, Z. & Kelly, P. J. Electrostatic doping of graphene through ultrathin hexagonal boron nitride films. *Nano Lett.* **11**, 4631–4635 (2011).

[6] Bardeen, J. Tunnelling from a many-particle point of view. *Phys. Rev. Lett.* **6**, 57–59 (1961).

[7] Duke, C. B. *Tunneling in solids*, vol. 10 (Academic Press, New York, 1969).

[8] Zhang, Y., Brar, V. W., Girit, C., Zettl, A. & Crommie, M. F. Origin of spatial charge inhomogeneity in graphene. *Nature Phys.* **5**, 722 (2009).

[9] Decker, R. *et al.* Local electronic properties of graphene on a bn substrate via scanning tunneling microscopy. *Nano Lett.* **11**, 2291–2295 (2011).

[10] Kresse, G. & Furthmüller, J. Efficient iterative schemes for ab initio total-energy calculations using a plane-wave basis set. *Phys. Rev. B* **54**, 11169 (1996).

[11] Perdew, J. P., Burke, K. & Ernzerhof, M. Generalized gradient approximation made simple. *Phys. Rev. Lett.* **77**, 3865 (1996).

[12] Grimme, S., Antony, J., Ehrlich, S. & Krieg, H. A consistent and accurate ab initio parametrization of density functional dispersion correction (dft-d) for the 94 elements h-pu. *J. Chem. Phys.* **132**, 154104 (2010).

[13] Marzari, N. & Vanderbilt, D. Maximally localized generalized wannier functions for composite energy bands. *Phys. Rev. B* **56**, 12847 (1997).

[14] Szabó, Á. *Dissipative quantum transport simulations in two-dimensional semiconductor devices from first principles.* Ph.D. thesis, ETH Zurich (2016).

[15] Luisier, M., Schenk, A., Fichtner, W. & Klimeck, G. Atomistic simulation of nanowires in the s p 3 d 5 s* tight-binding formalism: From boundary conditions to strain calculations. *Phys. Rev. B* **74**, 205323 (2006).

[16] Novotny, L. & Hecht, B. *Principles of Nano-Optics* (Cambridge University Press, Cambridge, 2012), second edn.

[17] Chew, W. C. *Waves and Fields in Inhomogeneous Media* (Van Nostrand Reinhold, New York, 1990), 1st edn.

[18] Koppens, F. H., Chang, D. E. & Garcia de Abajo, F. J. Graphene plasmonics: a platform for strong light–matter interactions. *Nano Lett.* **11**, 3370–3377 (2011).

[19] Falkovsky, L. A. & Varlamov, A. A. Space-time dispersion of graphene conductivity. *Eur. Phys. J. B* **56**, 281–284 (2007).

[20] Svintsov, D., Vyurkov, V., Ryzhii, V. & Otsuji, T. Voltage-controlled surface plasmon-polaritons in double graphene layer structures. *J. Appl. Phys.* **113**, 053701 (2013).

[21] Svintsov, D., Devizorova, Z., Otsuji, T. & Ryzhii, V. Plasmons in tunnel-coupled graphene layers: Backward waves with quantum cascade gain. *Phys. Rev. B* **94**, 115301 (2016).

[22] de Vega, S. & Garcia de Abajo, F. J. Plasmon generation through electron tunneling in graphene. *ACS Photonics* **4**, 2367–2375 (2017).

[23] Emani, N. K., Kildishev, A. V., Shalaev, V. M. & Boltasseva, A. Graphene: a dynamic platform for electrical control of plasmonic resonance. *Nanophotonics* **4**, 214–223 (2015).

[24] Adachi, S. *Optical constants of crystalline and amorphous semiconductors: numerical data and graphical information* (Springer Science & Business Media, 1999).

[25] König, T. A. *et al.* Electrically tunable plasmonic behavior of nanocube–polymer nanomaterials induced by a redox-active electrochromic polymer. *ACS Nano* **8**, 6182–6192 (2014).

[26] Johnson, P. B. & Christy, R. W. Optical constants of the noble metals. *Phys. Rev. B* **6**, 4370–4379 (1972).

[27] McPeak, K. M. *et al.* Plasmonic films can easily be better: rules and recipes. *ACS Photonics* **2**, 326–333 (2015).
